# The KSHV K1 Protein Modulates AMPK Function to Enhance Cell Survival

**DOI:** 10.1371/journal.ppat.1005985

**Published:** 2016-11-09

**Authors:** Penny M. Anders, Zhigang Zhang, Prasana M. Bhende, Louise Giffin, Blossom Damania

**Affiliations:** 1 Lineberger Comprehensive Cancer Center, University of North Carolina at Chapel Hill, Chapel Hill, North Carolina, United States of America; 2 Department of Microbiology and Immunology, University of North Carolina at Chapel Hill, Chapel Hill, North Carolina, United States of America; Hannover Medical School, GERMANY

## Abstract

Kaposi’s sarcoma herpesvirus (KSHV) is the etiologic agent of Kaposi’s sarcoma (KS) as well as two lymphoproliferative diseases, primary effusion lymphoma and multicentric Castleman’s disease. KSHV encodes viral proteins, such as K1, that alter signaling pathways involved in cell survival. Expression of K1 has been reported to transform rodent fibroblasts, and K1 transgenic mice develop multiple tumors, suggesting that K1 has an important role in KSHV pathogenesis. We found that cells infected with a KSHV virus containing a WT K1 gene had a survival advantage under conditions of nutrient deprivation compared to cells infected with KSHV K1 mutant viruses. 5’ adenosine monophosphate-activated protein kinase (AMPK) responds to nutrient deprivation by maintaining energy homeostasis, and AMPK signaling has been shown to promote cell survival in various types of cancers. Under conditions of AMPK inhibition, we also observed that cells infected with KSHV containing a WT K1 gene had a survival advantage compared to KSHV K1 mutant virus infected cells. To explore the underpinnings of this phenotype, we identified K1-associated cellular proteins by tandem affinity purification and mass spectrometry. We found that the KSHV K1 protein associates with the gamma subunit of AMPK (AMPKγ1). We corroborated this finding by independently confirming that K1 co-immunoprecipitates with AMPKγ1. Co-immunoprecipitations of wild-type K1 (K1_WT_) or K1 domain mutants and AMPKγ1, revealed that the K1 N-terminus is important for the association between K1 and AMPKγ1. We propose that the KSHV K1 protein promotes cell survival via its association with AMPKγ1 following exposure to stress.

## Author Summary

Infectious agents such as Kaposi’s sarcoma associated herpesvirus (KSHV) are etiologic agents of human cancer. KSHV-infected cells must survive various environmental stresses. Cells infected with KSHV express viral proteins that alter normal cellular processes to promote cell survival and viral persistence. We found that the KSHV K1 protein promotes survival under conditions of cellular stress, and that this survival advantage is at least partially dependent on the association of K1 and the cellular protein AMP-activated protein kinase (AMPK). We also observed increased AMPK activity in K1-expressing cells compared to EV following exposure to metabolic stress. Several reports suggest that AMPK signaling may contribute to tumor development by promoting cell survival. Our results suggest that KSHV K1 modulates cellular AMPK function to enhance the survival of KSHV-infected cells in order to promote viral persistence.

## Introduction

Kaposi’s sarcoma-associated herpesvirus (KSHV) is the causative agent of the endothelial cancer, Kaposi’s sarcoma (KS), and two B-cell lymphomas including primary effusion lymphoma (PEL) and multicentric Castleman’s disease (MCD) [1–3]. KSHV-related malignancies primarily arise in immune-suppressed individuals including HIV-positive individuals and organ transplant recipients, although these cancers can also occur in the absence of immunosuppression. KS is a common cancer in some sub-Saharan African countries [4, 5].

KSHV is a double-strand DNA gammaherpesvirus that is 165 to 170 kb long [6]. The KSHV genome contains multiple open reading frames that are conserved among other herpesviruses, and genes K1-K15 that are unique to KSHV [7]. Similar to other herpesviruses, KSHV has latent and lytic phases. Upon entering the host cell, KSHV typically establishes latency and expresses a limited number of viral proteins. Upon reactivation, which can be induced *in vitro* with various compounds such as 12-O-tetradecanoylphorbol-13-acetate (TPA), histone deacetylase (HDAC) inhibitors, and TLR 7/8 ligands, KSHV enters the viral lytic cycle resulting in the production of infectious virions [7, 8].

Both latent and lytic phases appear to be important for KSHV pathology. Expression of latent genes generally promotes the survival of the infected cell and persistence of infection during cell division. Lytic gene expression results in the production of inflammatory cytokines, pro-angiogenic factors and viral proteins that subvert the host immune system and promote virion production. KSHV K1 is primarily expressed during the lytic phase although recent studies indicate that K1 is also expressed at low levels during latency [9–11].

K1 is a 46-kDa transmembrane glycoprotein that contains a C-terminal immunoreceptor tyrosine-based activation motif (ITAM) analogous to the signaling molecules in the B-cell receptor (BCR) signaling complex [12]. The K1 ITAM has been found to interact with various SH2 containing signaling molecules, including among others, the p85 regulatory unit of phosphoinositide-3-kinase (PI3K) [13]. K1 has been shown to initiate a signaling cascade leading to intracellular calcium mobilization, upregulation of NFAT and AP-1 transcription factors, and production of inflammatory cytokines [12, 13]. It is thought that K1 is maintained in an activated state by oligomerization of the K1 ectodomain and subsequent phosphorylation of the ITAM tyrosines by Src family kinases [14].

K1 has a role in KSHV-induced tumor development. K1 expression immortalizes primary endothelial cells, transforms rodent fibroblasts, and K1 transgenic mice develop spindle cell sarcomatoid tumors and plasmablastic lymphoma, suggesting that the K1 protein is important for KSHV-induced tumor development [15–17]. These cancerous phenotypes may be due to K1’s modulation of cellular proteins in signaling pathways that are important for cell survival. We and others have previously shown that K1 activates the PI3K/Akt/mTOR pathway and protects against Fas-mediated apoptosis [18–20].

In our current studies, we observed that cells infected with KSHV viruses containing a wild-type K1 gene (KSHV-K1_WT_ and KSHV-K1_REV_) displayed a survival advantage under conditions of nutrient deprivation compared to viruses containing mutant K1 genes (KSHV-K1_5XSTOP_ and KSHVΔK1). To understand the underpinnings of this phenotype, we performed tandem affinity purification and mass spectrometry to identify K1 binding proteins. We found that KSHV K1 associates with the gamma subunit of 5’adenosine monophosphate-activated protein kinase (AMPKγ1).

AMPK is a heterotrimeric serine/threonine kinase composed of an alpha catalytic subunit and two regulatory subunits, beta and gamma [21]. Each subunit is part of a larger isoform family including the following subunit isoforms: α1, α2, β1, β2, γ1, γ2 and γ3 [22–25]. The isoforms of each subunit are found in different compartments within the cell. AMPKα1 and AMPKα2 localize to the cytoplasm. AMPKα2 also localizes to the nucleus in rat pancreatic and HeLa cells [26]. AMPKα1 and AMPKβ1 are in the perinuclear region in HEK-293 cells [27]. Mammalian AMPKα2, AMPKβ1, and AMPKγ1 are in the nuclei of neurons [28]. The subunit isoforms can come together in various combinations to make different heterotrimers. The differences in function of each heterotrimer are still under investigation. The presence of the three subunits is necessary for full activation of AMPK and the regulatory subunits stabilize expression of the catalytic α subunit [29].

AMPK responds to stresses that reduce ATP levels by inhibiting anabolic and activating catabolic pathways to maintain energy homeostasis [30]. Binding of adenosine monophosphate (AMP) to the gamma subunit allosterically activates AMPK and promotes phosphorylation of AMPKα at Thr^172^ by upstream kinases [31–33]. AMPK also responds to environmental stress factors that reduce cellular ATP levels such as hypoxia [34–37].

The role of AMPK as a tumor promoter is actively being explored [38, 39]. Some studies suggest that AMPK promotes tumor cell survival *in vitro* and *in vivo*. Inhibition of AMPK results in reduced prostate cell survival and increased apoptosis under normal and stressed conditions [40, 41]. AMPK has also been shown to promote survival in multiple myeloma, colorectal and glioma cancer cell lines [42–44]. *In vivo*, AMPK signaling was found to be elevated in developing tumors in a glioblastoma rat model [45]. Moreover, there is reduced *in vivo* tumor growth of xenografts prepared from transformed AMPKα1/α2-null MEFs compared to wild-type (WT) MEFs [37]. Thus, there is accumulating evidence suggesting that AMPK may promote cancer cell survival and tumor development.

Here we report that K1 binds AMPKγ1 and that this interaction is important for K1’s ability to enhance cell survival.

## Results

### Cells infected with KSHV containing WT K1 display increased survival

BAC16 recombinant viruses containing WT K1 (KSHV-K1_WT_ and KSHV-K1_REV_) were made as previously described [46]. Immortalized human umbilical vein endothelial cells (HUVEC) [17] or iSLK cells were infected with BAC16 recombinant viruses containing WT K1 (KSHV-K1_WT_ and KSHV-K1_REV_) or mutant K1 (KSHV-K1_5XSTOP_ and KSHVΔK1) genes [46]. These recombinant BAC16 viruses contain a GFP marker to monitor cell infectivity [46]. Both HUVEC and iSLK cells were stably selected until 100% of cells were green indicating that all cells were infected with these viruses.

**Fig 1:**
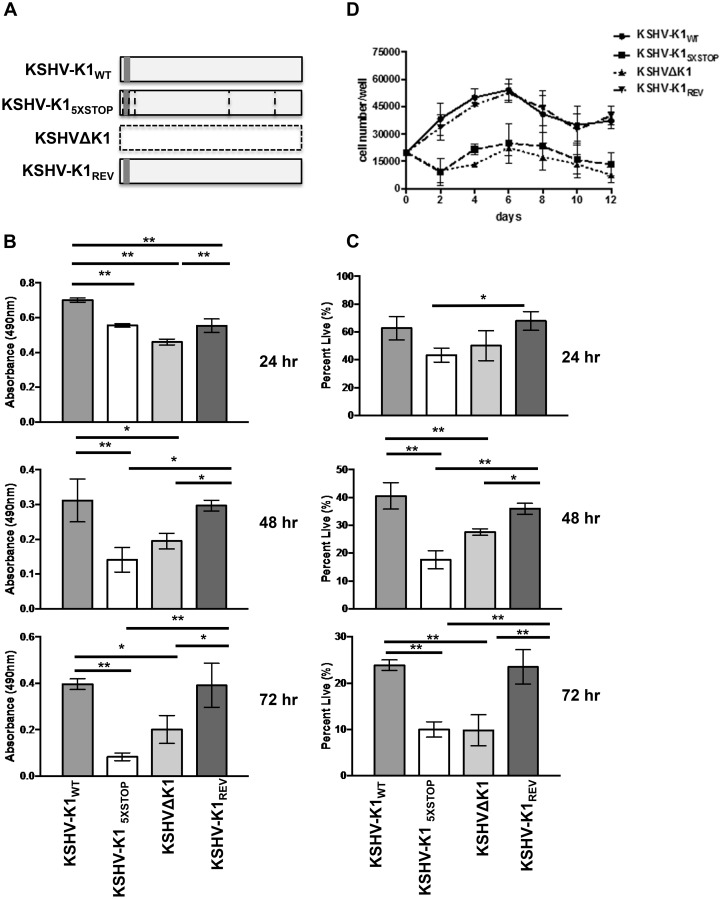
KSHV K1 mutant infected cells exhibit decreased survival following nutrient deprivation. (A) KSHV-K1_WT_ and KSHV-K1_REV_ (revertant) harbor a wild type K1 gene. The KSHV-K1_5XSTOP_ has a K1 mutant gene that contains 5 stop codons. Three of the stop codons follow the first start codon of the K1 gene. The other two stop codons replace two downstream ATG codons at positions 481 and 763 in the K1 gene. The KSHVΔK1 mutant has had the K1 gene replaced by a RpsL-Neo cassette. Hence, the KSHV-K1_5XSTOP_ and KSHVΔK1 are not able to express any portion of the K1 protein. The grey box represents FLAG and horizontal lines represent the stop codons in KSHV-K1_5XSTOP._ HUVEC infected with KSHV-K1_WT_, KSHV-K1_5XSTOP_, KSHVΔK1 or KSHV-K1_REV_ were starved of serum and growth factors for 24, 48 and 72 hours. (B) The proportion of metabolically active cells within each group was determined using an MTS assay. Error bars are the standard deviation of biological triplicates. GraphPad Prism was used to determine one-way ANOVA and Tukey’s post-test. *P<0.05, **P<0.005 (C) The percent of viable cells/well for each group was determined by trypan blue exclusion assay. Percent was calculated by normalization to total number of cells plated. Error bars are the standard deviation of biological triplicates. (D) KSHV-K1_WT_, KSHV-K1_5XSTOP_, KSHVΔK1, and KSHV-K1_REV_ infected iSLK cells were cultured without serum. Cell viability was determined by trypan blue exclusion. Error bars are the standard deviation of biological triplicates.

Each HUVEC cell line was then subjected to stress by withdrawing serum and growth factors. KSHV-K1_WT_ and KSHV-K1_REV_ (revertant) harbor a wild type K1 gene while the KSHV-K1_5XSTOP_ and KSHVΔK1 lack K1 (Fig 1A). We evaluated cell viability at various time-points by MTS ([3-(4,5-dimethylthiazol-2-yl)-5-(3-carboxymethoxyphenyl)-2-(4-sulfophenyl)-2H-tetrazolium, inner salt), which is a measure of metabolic activity, and trypan blue exclusion assay. At 24, 48, and 72 hours following nutrient deprivation, HUVEC infected with KSHV-K1_WT_ and KSHV-K1_REV_ had more viable cells compared to the KSHV-K1_5XSTOP_ and KSHVΔK1 HUVEC as observed using the MTS assay (Fig 1B). The differences were greatest at 72 hours post-nutrient deprivation.

To further substantiate these results, we also performed trypan blue exclusion assays and observed that the KSHV-K1_WT_ and KSHV-K1_REV_ infected cells were more viable compared to the cells infected with KSHV-K1_5XSTOP_ and KSHVΔK1 infected cells at 48 and 72 hours (Fig 1C). Similar to the MTS assay results, we observed reductions in cell viability in KSHV-K1_5XSTOP_ and KSHVΔK1 infected cells compared to KSHV-K1_WT_ and KSHV-K1_REV_ infected cells at 24, 48, and 72 hours post-starvation (Fig 1C). When we added serum and growth factors back to the media of KSHV-K1 WT and K1 mutant infected cells that had been starved for 72 hours, we noticed that there was still a reduction in viable KSHV-K1 mutant infected cells compared to the number of viable KSHV-K1 WT infected cells by trypan blue exclusion assay (S1B Fig). There is an increase in viable KSHV-K1 mutant cells from 72 hours of starvation to 72 hours of nutrient replenishment, just not to the same levels observed in KSHV-K1 WT (S1A and S1B Fig).

Furthermore, iSLK cell lines stably infected with these same viruses (KSHV-K1_WT_, KSHV-K1_REV_, KSHV-K1_5XSTOP_ and KSHVΔK1) were also evaluated following serum withdrawal. Every two days for a total of 12 days, we evaluated cell viability by trypan blue exclusion. At all time-points following serum withdrawal, we observed that KSHV-KT_WT_ and KSHV-K1_REV_ infected iSLK cells had increased viability compared to KSHV-K1_5XSTOP_ and KSHVΔK1 iSLK cells (Fig 1D). These findings suggest that K1 promotes KSHV-infected cell survival in the context of the whole genome and under conditions of nutrient deprivation.

A major regulator of metabolic stress is AMP-activated protein kinase (AMPK). AMPK responds to metabolic stress by activating catabolic pathways and inhibiting anabolic cell signaling pathways to maintain energy homeostasis [47]. Because we observed that cells infected with KSHV expressing a WT K1 gene had a survival advantage compared to cells infected with KSHV K1 mutant viruses following exposure to metabolic stress, and AMPK is involved in maintaining metabolic homeostasis, we hypothesized that K1 might modulate AMPK function. To explore this possibility, we compared cell survival in HUVEC infected with KSHV-K1_WT_ and KSHV-K1_REV_ wild-type viruses to KSHV-K1_5XSTOP_ and KSHVΔK1 mutant viruses following treatment with the AMPK inhibitor, compound C. Compound C is a reversible and competitive inhibitor of ATP [48]. In the presence of 5μM ATP and absence of AMP, compound C has a K_i_ of 109 ± 16 nM [48]. Compound C significantly prevents AMPK activation *in vitro* at 20 μM, and at 40 μM in cells treated with the AMPK activators metformin or AICAR [48]. According to Zhou et al., compound C has minimal impact on structurally related kinases such as ZAPK, SYK, PKCθ, PKA and JAK3 [48]. Inhibition of AMPK by compound C has been shown to induce cell death in various cell lines [41, 49].

**Fig 2:**
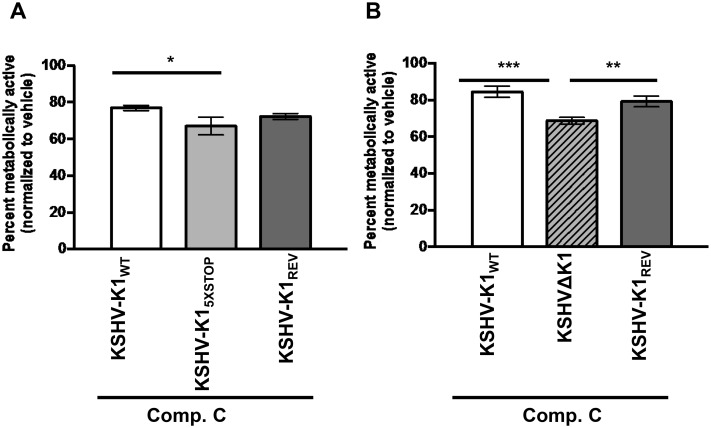
Cells infected with KSHV containing WT K1 are more resistant to AMPK inhibition than cells infected with KSHV K1 mutants. HUVEC infected with viruses containing a WT or mutant K1 gene were treated with 20 μM of compound C for 48 hours. Metabolically active cells were determined by an MTS assay. (A) The percent of metabolically active cells in HUVEC stably infected with the recombinant viruses KSHV-K1_WT_, KSHV-K1_5XSTOP_, and KSHV-K1_REV_. (B) The percent of metabolically active cells of HUVEC stably infected with KSHV- K1_WT_, KSHVΔK1, and KSHV-K1_REV._ The percent was determined by normalizing to DMSO (0.2%) control. Error bars represent the standard deviation of biological triplicates. GraphPad Prism was used to determine one-way ANOVA and Tukey’s post-test. *P<0.02, **P<0.01, ***P<0.001.

We observed increased cell viability in KSHV-K1_WT_ and KSHV-K1_REV_ infected cells compared to KSHV-K1_5XSTOP_ infected cells by MTS assay (Fig 2A). Corroborating these results, we also observed increased cell viability in KSHV-K1_WT_ and KSHV-K1_REV_ compared to KSHVΔK1 infected cells, suggesting that cells infected with WT K1 virus are less sensitive to the stress induced by AMPK inhibition than cells infected with the KSHV K1 mutants (Fig 2B). As expected, cell viability between KSHV-K1_WT_ and KSHV-K1_REV_ infected cells was essentially the same (Fig 2A and 2B).

To determine whether KSHV-K1_WT_ survival could be impacted by knock down of AMPK, we treated HUVEC stably infected with KSHV-K1_WT_ with AMPKα1 and AMPKα2 siRNA. We observed reduced cell viability in HUVEC infected with KSHV-K1_WT_ that had been treated with AMPKα1 and AMPKα2 siRNA compared to cells treated with NS siRNA at 48 and 72 hours (S2A Fig). We also evaluated AMPKα1 and AMPKα2 expression by immunoblot to confirm knock down of AMPKα1and AMPKα2 (S2B Fig). This data suggests that when AMPK is depleted, KSHV-K1_WT_ cells are susceptible to cell death.

**Fig 3:**
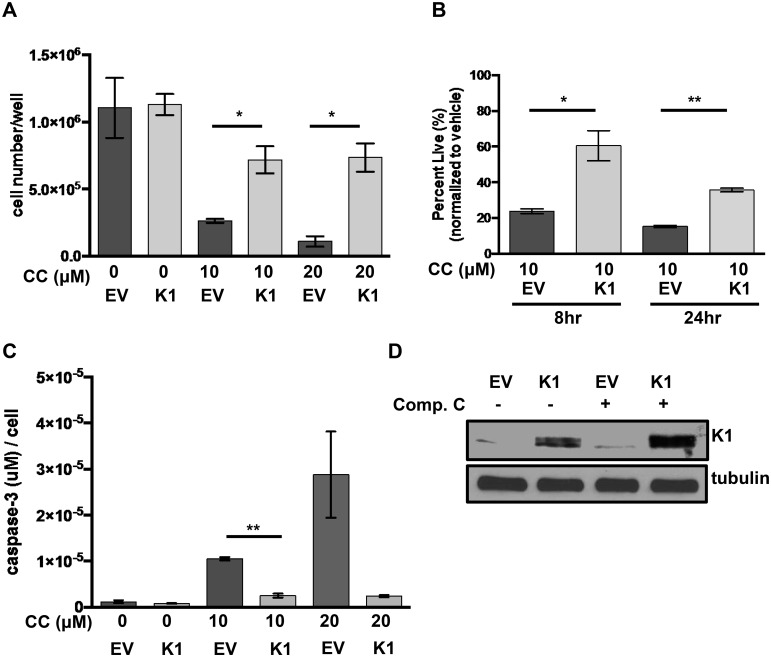
K1 expression provides a survival advantage in cells treated with compound C. (A) HEK-293 cells stably expressing empty vector (EV) or FLAG-K1 (K1) were treated in duplicate with 0 μM/ DMSO (0.2%), 20 μM compound C (CC) or 10 μM compound C for 6–8 hours. (B) HEK-293 cells stably expressing EV or K1 were treated in duplicate with 0 μM/DMSO (0.1%) or 10 μM compound C (CC) for 8 and 24 hours. Cell viability was determined by trypan blue exclusion assay. Percentages were derived from normalization to vehicle treated samples. (C) The level of caspase-3 activity was determined in duplicate samples and normalized to cell number of each sample. (D) EV or K1 HEK-293 cells treated with either 0 μM/DMSO (0.1%) or 10 μM compound C for 6 hours and immunoblotted for K1 and tubulin. Statistical significance was evaluated by Student’s t test. *P<0.05, **P<0.005.

To evaluate the impact of K1 expression by itself on cell survival following treatment with the AMPK inhibitor, compound C, we created FLAG epitope-tagged K1 (K1) or empty vector (EV) HEK-293 stable cell lines. The EV or K1 HEK-293 cells were treated with increasing concentrations of compound C, and cell viability was evaluated by trypan blue exclusion assay. We observed an increased number of viable cells in the K1 expressing cells compared to EV expressing cells using two different concentrations (10 μM and 20 μM) of compound C (Fig 3A). We observed this difference at both 8 and 24 hours following incubation of the cells with 10 μM compound C (Fig 3B). In order to determine whether K1 protected from apoptosis induced by AMPK inhibition, we also performed an assay to detect active caspase-3, which is an indicator of apoptosis. Active caspase-3 levels were remarkably elevated in EV cells compared to K1 expressing cells, suggesting that K1 protects cells from apoptosis when AMPK is inhibited (Fig 3C). K1 expression in these cells was confirmed by a Western blot (Fig 3D). This data suggests that K1 expression can keep cells alive by diminishing the effects of AMPK inhibition.

### The KSHV K1 protein associates with the gamma subunit of AMPK (AMPKγ1)

To investigate how K1 may be promoting cell survival following exposure to metabolic stress, we wanted to determine cellular proteins associated with K1. We identified K1-associated cellular proteins by performing tandem affinity purification of K1 from HEK-293 cells and subjecting cellular proteins bound to K1 to mass spectrometry.

**Fig 4:**
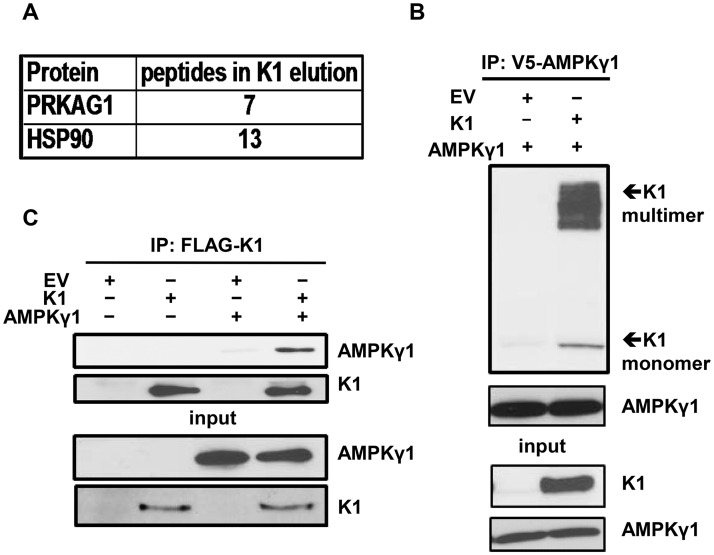
K1 associates with AMPKγ1. (A) Number of PRKAG1 (AMPKγ1) and HSP90 peptides identified in association with K1 by mass spectrometry. (B) HEK-293 cells stably expressing empty vector (EV) or FLAG-K1 (K1) were transfected with V5-AMPKγ1 (AMPKγ1). AMPKγ1 was immunoprecipitated, and immunoblotted for K1 or AMPKγ1. (C) HEK-293 cells stably expressing EV or K1 were transfected with AMPKγ1 or EV (pcDNA3). K1 was immunoprecipitated and immunoblotted for AMPKγ1 or K1.

Stable cell lines expressing a FLAG and HA double epitope-tagged version of K1 and EV HEK-293 cells were generated as previously described [50]. For tandem affinity purification, FLAG-HA-K1 or EV HEK-293 expressing cells were lysed with NP40 buffer. The clarified lysates were incubated with anti-FLAG M2 affinity gel, washed with NP40 buffer, and then eluted with 3X FLAG peptide. The eluates were subsequently incubated with an anti-HA resin, washed with NP40 buffer and eluted. The eluates were resolved by sulfate polyacrylamide gel electrophoresis (SDS-PAGE) and Coomassie stained. Only bands that were present in the K1 lane and absent in the EV lane were isolated and submitted for MALDI/TOF/TOF mass spectrometry (S3A Fig). A Western blot of the affinity-purified eluate was also performed to confirm successful pull-down of FLAG-HA-K1 (S3B Fig). Using mass spectrometry, we found AMPKγ1 associated with K1 (Fig 4A). We also observed an association between K1 and heat-shock protein 90 (HSP90) (Fig 4A), which confirmed our previous report on the association of K1 with HSP90 [50].

Next, we constructed a V5 epitope-tagged AMPKγ1 (AMPKγ1) in pcDNA3 vector. To confirm the association between K1 and AMPKγ1 as determined by mass spectrometry, we transiently expressed AMPKγ1 in HEK-293 cells stably expressing EV or FLAG epitope-tagged K1 (K1). We performed a co-immunoprecipitation by incubating EV- or K1-expressing HEK 293 clarified lysates containing equal amounts of protein with anti-V5 antibody to pull down the V5 epitope-tagged AMPKγ1. We detected the multimer and monomer forms of K1 co-immunoprecipitating with AMPKγ1 in K1-expressing cells, but not from EV control cells (Fig 4B). To further substantiate the association between K1 and AMPKγ1, we performed the reverse immunoprecipitation and immunoprecipitated K1. We observed that AMPKγ1 co-immunoprecipitated with K1 from K1-expressing cells but not from EV control cells (Fig 4C).

**Fig 5:**
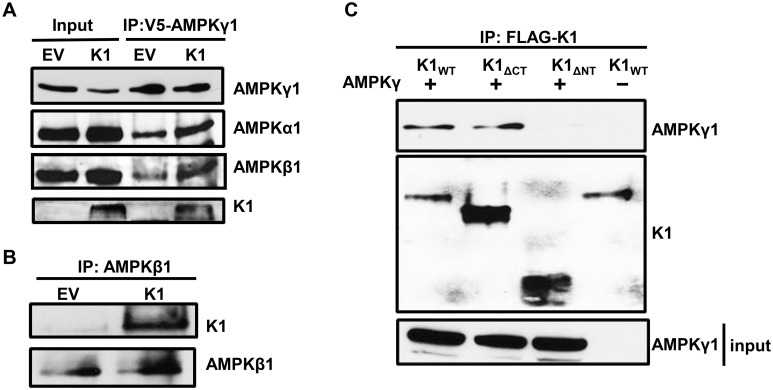
The K1 N-terminus is important for association with AMPKγ1. (A) HEK-293 cells stably expressing empty vector (EV) or FLAG-K1 (K1) were transfected with V5-AMPKγ1 (AMPKγ1). AMPKγ1 was immunoprecipitated, and blots were probed for AMPKγ1, endogenous AMPKα1, endogenous AMPKβ1 and K1. (B) Endogenous AMPKβ1 was immunoprecipitated from EV or K1 expressing HEK-293 cells. The blots were probed for K1 or AMPKβ1. (C) HEK-293 cells were transiently transfected with AMPKγ1 and with one of the following: K1_WT_, K1_ΔCT_ or K1_ΔNT_. An equivalent amount of EV (pcDNA3) was also transfected along with K1_WT_. We immunoprecipitated the various K1 domain mutants, and immunoblotted for AMPKγ1 or K1.

In the cell, AMPKγ1 complexes with AMPKα1 and AMPKβ1. In addition to AMPKγ1, we next wanted to determine whether the other AMPK subunits were part of the protein complex associated with K1. We transfected V5-AMPKγ1 in HEK-293 cells stably expressing empty vector (EV) or FLAG-K1 (K1), immunoprecipitated V5-AMPKγ1, and probed for endogenous AMPKα1 and AMPKβ1. In addition to detecting K1 as we previously observed, we also observed the expression of AMPKα1 and AMPKβ1 (Fig 5A), suggesting that there is an association between K1 and the three subunits of AMPK. Next, we determined whether we could detect association of K1 and endogenous AMPK. As the commercially available AMPKγ1 antibody is not appropriate for immunoprecipitation, we immunoprecipitated endogenous AMPKβ1 and probed for K1 (Fig 5B). Upon AMPKβ1 immunoprecipitation, we detected K1, which further confirmed an association between K1 and the endogenous AMPK complex (Fig 5B).

K1 has an immunoglobulin-like N-terminus, a transmembrane region, and a cytoplasmic tail containing an ITAM. To identify the region of K1 that associates with AMPKγ1, we performed co-immunoprecipitations of various FLAG-tagged K1 domain deletion mutants and V5-tagged AMPKγ1. We transiently expressed V5-AMPKγ1 (AMPKγ1) along with one of the following in HEK-293 cells: K1_WT_, K1 lacking the C-terminus (K1_ΔCT_), or lacking the N-terminus (K1_ΔNT_). We also transfected an equivalent amount of EV (pcDNA3) and K1_WT_ as a control. The construction of the FLAG-tagged K1 mutants has previously been described [50]. We immunoprecipitated K1_WT_, K1_ΔCT_, or K1_ΔNT_ and probed for V5-tagged AMPKγ1 (Fig 5C). As previously observed, we detected co-immunoprecipitation of AMPKγ1 and K1_WT_ (Fig 5C, lane 1). We also detected co-immunoprecipitation of AMPKγ1 and K1_ΔCT_ indicating that the K1 C-terminus is not important for K1 and AMPKγ1 association (Fig 5C, lane 2). We did not observe co-immunoprecipitation of AMPKγ1 and K1_ΔNT_ suggesting that AMPKγ1 associates with K1 via the K1 N-terminus (Fig 5C, lane 3).

### K1 and AMPK subunits are localized to cellular membranes

**Fig 6:**
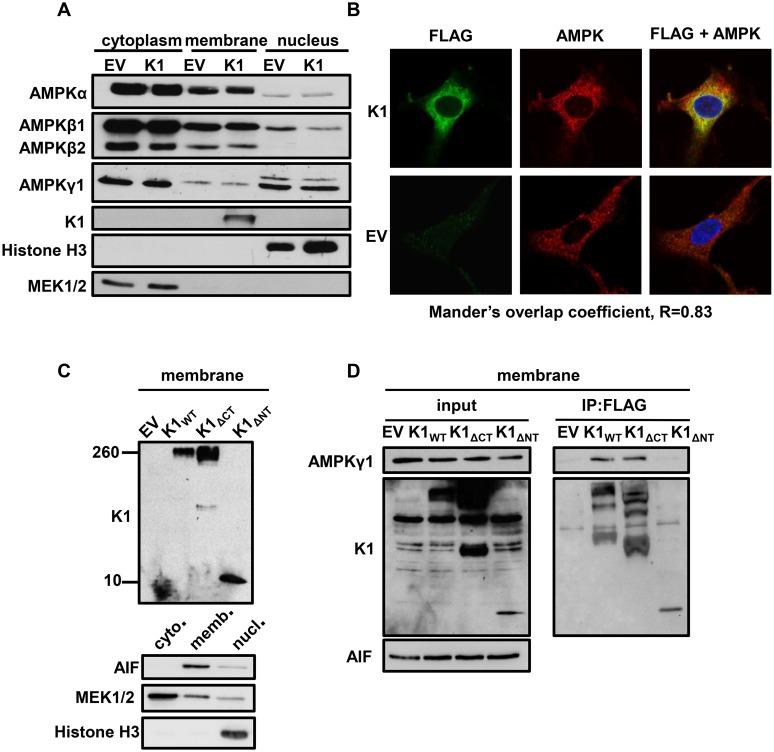
K1 and AMPK associate in membranes in the perinuclear area of the cell. (A) HEK-293 cells stably expressing empty vector (EV) or FLAG-K1 (K1) were lysed and fractionated into cytoplasmic, membrane and nuclear fractions. Immunoblots were probed for endogenous AMPK subunit and isoforms. (B) HUVEC stably expressing empty (EV) or FLAG-K1 (K1) were fixed and permeabilized. Cells were then stained with anti-FLAG directly conjugated to FITC antibody, and an AMPKβ1/2 antibody, followed by an anti-rabbit conjugated to Alexa Fluor 647. Nuclei were stained with DAPI. Stained HUVEC were evaluated with a Zeiss 700 confocal microscope using a 63X oil objective. Mander’s overlap coefficient (R = 0.83) was generated using ImageJ. (C) HEK-293 cells were transiently transfected with EV (pcDNA3), K1_WT_, K1_ΔCT_ and K1_ΔNT_. Cell lysates were separated into membrane, cytoplasmic and nuclear fractions. We evaluated the membrane fraction for expression of the K1 domain deleted mutants by immunoblot. We also immunoblotted for AIF (membrane), MEK1/2 (cytoplasm) and Histone H3 (nucleus). (D) HEK-293 cells were transiently transfected with the constructs described in (C) with the addition of AMPKγ1. Cell lysates were separated into membrane, cytoplasmic and nuclear fractions. We immunoprecipitated FLAG K1 and immunoblotted for AMPKγ1 or FLAG using only the membrane fraction.

About 10–20% of K1 is localized to the plasma membrane with the major fraction of K1 being found in the endoplasmic reticulum [51]. K1 can also be internalized and internalization is associated with K1 signaling [52]. Additionally, all three subunits of AMPK have been shown to localize to the cellular membrane fraction [27]. We wanted to evaluate the localization of the endogenous AMPK subunits in EV- and K1-expressing stable HEK-293 cells. We lysed equal numbers of EV- and K1-expressing HEK-293 cells and separated the cellular fractions. The fractions were then resolved by SDS-PAGE and Western blot. The blots were probed using antibodies specific for each AMPK subunit and isoform. In addition to being localized to the cytoplasm, we observed that AMPKα, AMPKβ1, and AMPKγ1 were detected in the cellular membrane fraction (Fig 6A). Along with the AMPK subunits, K1 was also found in the membrane fraction (Fig 6A). We evaluated the purity of the cytoplasmic, membrane, and nuclear fractions by probing for MEK1/2, K1, and histone H3 respectively (Fig 6A). These proteins are restricted to each of these fractions. Based on these findings, we conclude that K1 and multiple AMPK subunits and isoforms (without over expression) are localized to the cellular membrane fraction, suggesting that K1 associates with AMPK in the cellular membrane.

We subsequently evaluated localization of FLAG-K1 (K1) and endogenous AMPK by immunofluorescence staining. Because there is no available AMPKγ1 antibody that was appropriate for immunofluorescence staining, and we had determined that AMPKβ1 co-localized with K1 (Fig 5B), we stained for K1 and endogenous AMPKβ1/2 in EV- or K1- stably expressing HUVEC. We fixed the cells with formaldehyde, washed and then permeabilized the cells with Triton-X-100. We stained cells with FLAG-FITC to detect FLAG-K1 and AMPKβ1/2 antibodies followed by an anti-rabbit Alexa Fluor 647 secondary antibody. By confocal microscopy, we acquired z-stacks on fully stained EV and FLAG-tagged K1 HUVEC (Fig 6B). We also completed z-stacks on controls containing only the secondary anti-rabbit AF647 in order to demonstrate that the staining for AMPKβ1/2 is specific and not due to non-specific secondary staining. We observed co-localization of K1 and endogenous AMPKβ1/2 in the perinuclear area (Fig 6B) as determined by a Mander’s overlap coefficient of 0.83, which was determined using ImageJ software. The EV transfected cells stained positive for AMPKβ1/2 but not K1, as expected.

As described above, we observed (Fig 5C) that K1_ΔNT_ does not associate with AMPKγ1 by co-immunoprecipitation. This lack of association may be due to the fact that the two proteins do not interact or because K1_ΔNT_ is mislocalized in the cell. In order to confirm that K1_ΔNT_ is expressed in the membrane but still does not interact with AMPK, we transiently expressed K1_ΔNT_, and fractionated the cell lysates into cytoplasmic, membrane, and nuclear components. We then evaluated K1_ΔNT_ expression in the membrane fraction by immunoblotting for K1_ΔNT_. We observed that K1_ΔNT_ is expressed in the membrane fraction (Fig 6C). Next, we transiently transfected the K1 domain deletion mutants and AMPKγ1. We separated the lysates into membrane, cytoplasmic, and nuclear fractions. We removed detergent from the membrane fraction by spin column removal and completed a bicinchoninic acid assay (BCA assay) to determine the protein concentrations for each sample. Using equal amounts of protein, we immunoprecipitated EV (pcDNA3), K1_WT_, K1_ΔCT_, or K1_ΔNT_ and probed for V5-tagged AMPKγ1 in the membrane fraction. We detected K1_WT_ and AMPKγ1 association, but we did not detect K1_ΔNT_ and AMPKγ1 association in the membrane fraction (Fig 6D) corroborating our previous findings.

### K1 and AMPK association is important for cell survival following exposure to stress

**Fig 7:**
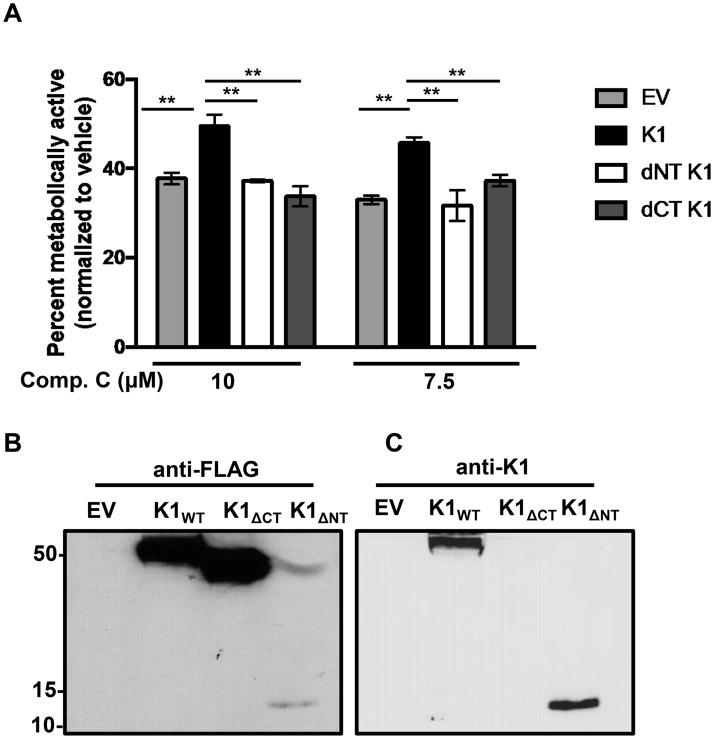
K1 and AMPK association is important for cell survival following exposure to stress. (A) HEK-293 cells stably expressing FLAG-tagged K1_WT_, K1_ΔCT_, K1_ΔNT_ or empty vector (EV = pLenti CMV) were treated with 10 or 7.5 μM compound C for 48 hours. The percent of metabolically active cells was determined using the Promega MTS assay. The percent was derived by normalization to the DMSO (0.1%) control. Error bars represent the standard deviation of biological triplicates. GraphPad Prism was used to determine one-way ANOVA and Tukey’s post-test. **P<0.005 (B) Western blot showing expression of EV, K1_WT_, K1_ΔCT_, and K1_ΔNT_ using an anti-FLAG antibody. (C) Western blot from (Fig 7B) was stripped and probed for K1 using an anti-K1 antibody.

Thus far, we have observed that KSHV K1 promotes survival in stressed cells, and K1 associates with AMPK via the K1 N-terminus. We next wanted to determine whether the association of K1 and AMPK is important for the survival advantage observed in stressed cells. We generated lentivirally transduced HEK-293 cells stably expressing FLAG epitope-tagged K1_WT_, K1_ΔCT_, K1_ΔNT_ and empty vector (EV). We treated these cells with the AMPK inhibitor, compound C, and evaluated cell viability using the MTS assay. We observed an increased percentage of viable K1_WT_ expressing cells when AMPK was inhibited, compared to cells expressing K1_ΔNT_. This result suggests that the association between K1 and AMPK is important for survival in stressed cells (Fig 7A). Surprisingly, we also observed that K1_ΔCT_ expressing cells appear sensitive to AMPK inhibition, indicating that the K1-C terminus is also important for survival in stressed cells (Fig 7A). We confirmed expression of the various K1 constructs by completing a Western blot using an anti-FLAG antibody or an anti-K1 antibody (Fig 7B). When we immunoblotted with an anti-FLAG antibody, we observed low levels of K1_ΔNT_. Thus, we re-probed this blot using an anti-K1 antibody, and saw expression of K1_ΔNT_ but no K1_ΔCT_ expression since the K1 antibody is directed towards an epitope on the K1 C-terminus (Fig 7C).

### K1 facilitates AMPK activity in stressed cells

**Fig 8:**
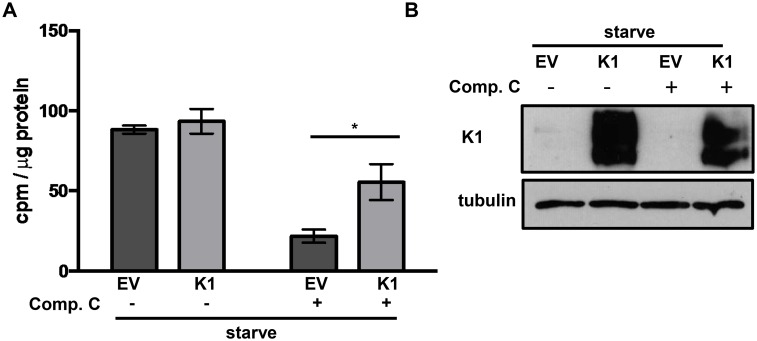
K1-expression facilitates AMPK activity in stressed cells. (A) HUVEC stably expressing empty vector (EV) or FLAG-K1 (K1) were deprived of serum and growth factors (starve) for 24 hours. At the time of starvation, 5 μM compound C or DMSO (0.05%) was added. Each condition for EV and K1 was performed in triplicate. AMPK-specific activity was determined by subtracting counts per minute (cpm) derived for each sample incubated without SAMS peptide from cpm derived from each sample incubated with SAMS peptide. These values were then normalized to total protein for each sample. The error bars represent the standard deviation of triplicates. *P<0.05 Student’s T test. (B) Western blot of K1 and tubulin using lysate from the AMPK activity assay.

We found that K1 associates with AMPK and this association is important for the survival advantage in stressed cells. We next wanted to determine the status of AMPK activity in stressed EV and K1 expressing cells. We exposed HUVEC stably expressing empty vector (EV) or FLAG-tagged K1 (K1) to media without serum and growth factors containing either compound C or DMSO control for 24 hours. We then performed an AMPK-specific kinase activity assay. We incubated lysate from EV or K1 expressing HUVEC with or without an AMPK substrate, a synthetic peptide called SAMS peptide (HMRSAMSGLHLVKRR), AMP, and radiolabeled γ-32P-ATP [53]. We next evaluated the incorporation of radiolabeled phosphate from γ-32P-ATP into SAMS peptide. Compound C treatment resulted in an overall reduction in AMPK activity in both EV and K1 expressing cells compared to untreated cells. However, in the presence of compound C, we observed a higher degree of AMPK activity in K1 expressing cells compared to EV expressing cells (Fig 8A). This data suggests that K1 expression promotes AMPK activity in stressed cells. We also confirmed that K1 expression is not altered by nutrient deprivation and compound C treatment by Western blot analysis (Fig 8B).

## Discussion

Cell survival during KSHV infection is paramount to the establishment of life-long infection. Upon infection, KSHV primarily enters a latent state. During latency, KSHV expresses a limited number of proteins and microRNAs that enable it to successfully persist in the cell by avoiding the immune response and by promoting cell survival [54]. When KSHV reactivates and enters the lytic stage, the cell must remain viable throughout viral replication and virion assembly so that infectious virions are generated. Cell death prior to completion of the lytic program would result in defective viral replication.

We observed that KSHV infected cells containing a WT K1 gene had a survival advantage compared to cells infected with KSHV K1 mutants following exposure to stress. To explore the underpinnings of this phenotype, we completed tandem affinity purification and mass spectrometry to identify K1-associating proteins. We identified AMPKγ1 as a K1-associating protein. We corroborated this finding independently and found that K1 co-immunoprecipitated with AMPKγ1. By performing co-immunoprecipitations of AMPKγ1 and K1 domain mutants, we found that the K1 N-terminus is important for K1 and AMPKγ1 association. The association between K1 and AMPK is important for the survival advantage in stressed cells because we observed reduced cell viability in cells expressing K1_ΔNT_ compared to K1_WT_. Our studies indicate that KSHV K1 promotes survival via its association with AMPK, and KSHV K1 facilitates AMPK activity in stressed cells.

To explore alternative possibilities for not observing the association between K1_ΔNT_ and AMPKγ1, we confirmed that K1_ΔNT_ is not mislocalized in the cell and is expressed in the membrane by performing a Western blot for K1_ΔNT_ in the membrane fraction. Another explanation for not observing association between AMPKγ1 and K1_ΔNT_ may be that K1_ΔNT_ may not fold correctly due to the lack of the N-terminus.

Under normal culture conditions, our lab and others have shown that K1 activates the PI3K/Akt/mTOR pathway [13, 18]. It has been reported that cells having overly active Akt and consequently a high glycolytic rate are more sensitive to cell death following starvation compared to control cells [55]. Moreover, when starved-cells are treated with an activator of AMPK, cells with active Akt are protected from cell death [55]. KSHV-infected cells also have an active PI3K/Akt/mTOR pathway and a high glycolytic rate [56–58]. Furthermore, simultaneous activation of AMPK, Akt and mTOR, has been observed in liver cancer cells following nutrient starvation [59]. Thus, low levels of AMPK activation may promote metabolic adaptation and consequently, increase KSHV-infected cell survival. We propose that K1 promotes AMPK activity during metabolic stress and in this way enhances KSHV-infected cell survival.

We observed a modest reduction in the number of viable KSHV-K1_WT_ cells following knock down of the catalytic subunits, AMPKα1 and AMPKα2, compared to KSHV-K1_WT_ cells treated with non-specific siRNA (S2A Fig). This data suggests that in addition to promoting cell survival via AMPK, K1 likely contributes to the survival advantage observed in KSHV-K1_WT_ cells by activating other pro-survival pathways such as the PI3K/Akt pathway as has been previously described [18] [19, 20]. Thus, K1 promotes cell survival by multiple methods, including its association with AMPKγ1.

One of the K1-interacting proteins previously identified is Syk, a Src homology 2 (SH2)-containing protein tyrosine kinase [13]. Activation of Syk in B cell lymphomas is associated with cell survival since inhibition of Syk is clinically efficacious in treating lymphoma [60]. Moreover, Syk activity is critical for the normal functioning of endothelial cells and vascular integrity *in vivo*. Syk deficient mice exhibit petechiae in utero, as well as, a reduced number of endothelial cells that are morphologically defective, indicating that Syk activity is critical for endothelial cell survival and function [61]. *In vitro*, Syk activity is important for HUVEC proliferation and migration [62]. Huang et al. found that AMPK associates with Syk and induced its activation [63]. Although the association of AMPK and Syk has not been evaluated in HUVEC, we may speculate that the association of K1, AMPK, and Syk may facilitate downstream signaling cascades that promote cell survival.

The role of AMPK during herpesvirus infection is complicated, and whether it promotes viral replication or inhibits it may depend on a variety of factors. During human cytomegalovirus (HCMV) infection, AMPK has been found to promote a metabolic environment that is conducive to viral replication [64, 65]. HCMV infection augments glycolysis and AMPK inhibition blocks increased glycolysis that is induced by HCMV infection and AMPK inhibition also hinders viral DNA synthesis [65]. During human herpes simplex virus-1 (HSV-1) infection, AMPK activity facilitates neuron survival and reduces viral production [66]. Thus, AMPK appears to impact viral production differently during HCMV and HSV-1 infection.

Recently, Cheng et al. observed that endogenous AMPKα1 inhibits KSHV replication following primary infection [67]. AMPK does not seem to affect KSHV infectivity nor trafficking to the nucleus, but it does have an inhibitory effect on KSHV lytic gene expression since knockdown of AMPK results in increased expression of some KSHV lytic genes and corresponding proteins [67]. Thus, AMPK appeared to inhibit the KSHV lytic cycle but not the establishment of latency.

Our studies examine the role of AMPK in a different context i.e. in latently infected cells expressing WT or mutant K1 under conditions of metabolic stress. We report that AMPK activity contributes to survival of latent KSHV-infected cells. Because AMPK can be activated by a variety of cellular stresses and can impact multiple cell signaling pathways, it is highly plausible that AMPK can differentially impact the infected-cell depending on the life-cycle of the virus. There are also multiple isoforms of AMPK, and various combinations of these isoforms can form different heterotrimeric complexes. We do not yet understand how these different AMPK combinations modulate AMPK function, and this is an additional complexity that we need to understand in the future in order to evaluate AMPK’s function during the KSHV lifecycle.

## Materials and Methods

### Cell culture, transfections, and chemical compounds

HEK-293 (ATCC, CRL-1573) and iSLK cells [46] were maintained in Dulbecco’s Modified Eagle Medium (DMEM) and human telomerase-reverse transcriptase-immortalized human umbilical vein endothelial cells (hTERT-HUVEC) [17] were cultured in endothelial growth basal medium (EBM-2) from Lonza and supplemented with an endothelial cell growth medium (EGM-2) bullet kit without heparin and ascorbic acid supplements. All cell lines were supplemented with 10% heat-inactivated fetal bovine serum (HI-FBS), 1% penicillin-streptomycin (PS), and 1% L-glutamine and maintained at 37°C and 5% CO_2_. Additionally, EV (pcDNA3) or FLAG-K1 stably expressing HEK-293 cells were maintained in 1 mg/mL G418. For transfection of HEK-293 cells, cells were transfected with 10 μg pcDNA3-V5-AMPKγ1 or vector per 10 cm plate using XtremeGENE HP reagent according to the manufacturer’s instructions. The AMPK inhibitor, compound C, was purchased from Calbiochem and suspended in dimethyl sulfoxide (DMSO).

### Constructs

HEK-293 cells stably expressing EV or FLAG-HA-K1 were made as previously described [50]. Briefly, FLAG-HA was cloned following the signal peptide sequence on the N-terminus of K1 (Accession# AAG01599.1). The FLAG-HA-K1 was then cloned into the pcDNA3 vector. A V5-epitope tag was added to the N-terminus of PRKAG1 (NP_002724) by PCR and then cloned into the pcDNA3 vector. The pcDNA3-K1 WT that had previously been made [68] was used as a template for preparing the K1 domain deleted mutants, which have been previously described [50]. To construct pcDNA3-FLAG-K1_ΔCT,_ pcDNA3-K1 WT (amino acids 1–303) and the following primers, for-5’-CGCCCGAAGCTTATGGCCCTGCCCGTGACCGCCCTG-3’ and rev-5’-CGCCACAAGGTTTCAGTACCAATCCACTGGTTG-3’ were combined for amplification by PCR. The PCR product was then cut with HIND III and cloned into pcDNA3. pcDNA3-FLAG-K1_ΔCT_ lacks the amino acids 266–303. To construct pcDNA3-K1_ΔNT_-FLAG, pcDNA3-K1 WT was combined with primers, for-5’-CATCTTGCATCCAGTATTTATGACAC-3’ and rev-5’-CGCCGCTCTAGATTCCACTGGTTGCG-3’. The PCR product was then cut with Bam HI and XbaI, and cloned into pcDNA3. pcDNA3-K1ΔNT−FLAG lacks amino acids 1–241. The pcDNA3-K1 WT construct was used as a template for making the lentiviral K1 WT and mutant constructs. pLenti-FLAG-K1 domain mutants were generated using Q5 Site-Directed Mutagenesis kit (Q5) by New England Biolabs Inc and appropriate primer sets were designed according to the Q5 kit specifications. FLAG-K1, FLAG-K1_ΔCT_, and FLAG-K1_ΔNT_ were cloned into the lentiviral vector, pLenti CMV Puro DEST. Empty vector is pLenti CMV Puro. DEST.

### Generation of stable cell lines

pcDNA3-FLAG-HA-K1 or pcDNA3 empty vector were transfected into HEK-293 cells and selected in media containing 1 mg/mL G418. All lentiviruses were prepared using the Virapower lentiviral expression system as per the manufacturer’s instructions (Invitrogen). hTERT-HUVEC or HEK-293 cells were cultured to approximately 80% confluency in a 6-well dish. At the time of lentiviral transduction, cells were rinsed with PBS and 2 mLs of lentivirus (unconcentrated) was added in the presence of 10 μg/mL polybrene. The cells were centrifuged for 90 minutes at 3000 RCF at 30°C. The cells were then incubated overnight at 37°C and 5% CO_2_. The media containing lentivirus was replaced with the appropriate fresh media the following day. Forty-eight hours following lentiviral transduction, HUVEC cells underwent selection with 0.5 μg/mL puromycin for 1 week. Transduced HEK-293 cells underwent selection with 1 μg/mL puromycin for 1–2 weeks.

### Tandem affinity purification

The FLAG HA Tandem Affinity Purification Kit by Sigma was used. FLAG-HA-K1 or EV HEK-293 expressing cells were lysed and the lysates were incubated with anti-FLAG M2 affinity gel, washed and eluted with 3X FLAG peptide. The eluates were subsequently incubated with an anti-HA resin, washed with NP40 buffer and eluted. The eluates were resolved by sulfate polyacrylamide gel electrophoresis (SDS-PAGE) and Coomassie stained. Bands present in the K1 sample but absent in the EV were submitted to the Harvard Mass Spectrometry core for MALD/TOF/TOF mass spectroscopy analysis.

### Trypan blue exclusion assay

EV or FLAG-HA-K1 stably expressing HEK-293 cells were plated at 650,000 cells/well in a 6-well dish or at 60,000/well in a 24-well dish. The next day, the media was removed and replaced with complete media containing the relevant concentration of compound C. Six to eight hours later the media from each well was collected; the cells were gently washed with PBS and trypsinized. The cells were pelleted and resuspended in 0.6–1 mL complete media. An aliquot was removed for the trypan blue exclusion assay. The cells were then pelleted, the supernatant was discarded and the pellets were immediately frozen and maintained at -80°C until utilized for the caspase-3 assay. HUVEC infected with KSHV containing WT K1 or mutant K1 were plated at 20,000 cells per well of a 24-well plate in EBM-2 without serum and growth factors. Cells were not previously washed. iSLK cells containing WT K1 or mutant K1 were plated in a similar manner, but in complete media. The next day, the iSLK media was replaced with serum-free DMEM. At the time of counting, cells were trypsinized and cell suspension was then combined with trypan blue (0.4% Sigma Aldrich) at a 1:1 dilution. Each sample was counted in duplicate or triplicate using a hemacytometer.

### Active caspase 3 assay

The previously frozen cell pellets were thawed on ice. The caspase-3 assay was then completed based on the manufacturer’s instructions (ApoAlert Caspase-3 Fluorescent Assay by Clontech Laboratories). Briefly, the pellets were lysed and maintained on ice followed by centrifugation. The clarified supernatant was then assayed for active caspase-3 and fluorescence was determined using the CLARIOstar plate reader (BMG Labtech). Active caspase-3 concentrations were determined using a standard curve, and active caspase-3 values were then further normalized to cell number.

### MTS assay

Five thousand cells per 100 μL were counted and resuspended in EBM-2 containing 30 μg/mL hygromycin but lacking all other supplements. Cells were plated in triplicate and incubated for 24, 48 and 72 hours. Cell proliferation was determined using the Cell Titer 96 Aqueous One Solution Cell Proliferation Assay (Promega) according to the manufacturer’s instructions. Stored aliquots of previously frozen MTS were thawed in a water bath at 37°C. Twenty microliters of MTS reagent was then dispensed into each well using a multichannel pipet. The plate was then gently shaken for 30 seconds and placed in an incubator at 37°C for 2–3 hours. At the end of incubation, the plate was gently tapped to mix the formazan product. The absorbance was then immediately measured at 490 nm using a CLARIOstar plate reader (BMG Labtech). Wells that contain only media were subtracted as background from all OD values. Elevated absorbance values are indicative of metabolically active cells. For compound C treatments, we normalized to vehicle treated cells. For the starvation of HUVEC, the same number of cells was plated. In these experiments OD values are not normalized but show relative optical density values.

### Cell fractionation

HEK-293 stably expressing EV (pcDNA3) or FLAG-HA-K1 (pcDNA3) were washed, trypsinized, centrifuged and counted. Five million cells were prepared using a cell fractionation kit according to the manufacturer’s instructions (Cell Signaling Technology). Equal volumes of each lysate from each fraction for EV and K1 were loaded and resolved by sodium dodecyl sulfate-polyacrylamide gel electrophoresis (SDS-PAGE) and then transferred to a nitrocellulose membrane.

### Immunoblots

Cells were harvested, washed twice with PBS, and then lysed in buffer containing 0.5% NP40, 150 mM NaCl, 50 mM Tris-HCL pH 8.0, and a cocktail of proteinase (Roche) and phosphatase (Roche) inhibitors. The lysates used to evaluate K1 protein expression were frozen and thawed two times. Protein concentrations were determined by Bradford assay. Equal amounts of protein (15–25 μg) were loaded per lane and resolved by SDS-PAGE and then transferred to a nitrocellulose membrane. The following antibodies from Cell Signaling Technology were used: AMPKα #2603, AMPKα1 #2795, AMPKα2 #2757, AMPKβ1 #12063, AMPKβ1/2 #4150, AMPKγ1#4187, HRP-linked anti-rabbit IgG #7074 and HRP-linked anti-mouse IgG #7076. In some experiments K1 expression was confirmed by immunoblotting with an HRP-conjugated mouse monoclonal anti-FLAG M2 antibody from Sigma #F1804. The K1 monoclonal antibody was made by immunization with the peptide, KQRDSNKTVP, protein ID#AAB71616 (gene accession #U86667).

### Co-immunoprecipitations

Lysates containing equal amounts of protein as determined by the Bradford assay or bicinchoninic acid assay (BCA assay) were combined with EZview Red anti-FLAG M2 affinity gel (Sigma, F2426) as per the manufacturer’s instructions. For V5-AMPKγ1 immunoprecipitation, protein A/G agarose (Santa Cruz, sc-2003) was combined with monoclonal anti-V5 antibody (Sigma, V8012), which was used at 1ug of antibody/1 mg of protein, and rotated overnight at 4°C. The supernatant was removed and the affinity gel or agarose pellets were washed by adding 1mL of 0.1% NP40 lysis buffer followed by 5 minutes rotation at 4°C for a total of 4 times. For immunoprecipitation using the membrane fraction, affinity gel pellets were washed with 1mL of 0.1% NP40 lysis buffer, followed by 5 minutes rotation at 4°C for 3 times and with 1mL 0.5% NP40 lysis buffer followed by 5 minutes rotation at 4°C for one time. Detergent was then removed from the membrane fraction using Pierce Detergent spin columns. FLAG-K1 and/or FLAG-K1 domain deleted mutants were eluted using 3X FLAG peptide (Sigma, F4799) as per the manufacturer’s instructions. Laemmli buffer (2X) was added to the FLAG-K1eluate or directly to the V5-AMPKγ1/agarose samples (1:1) and all samples were heated at 100°C for 6 minutes. Proteins were resolved by SDS-PAGE followed by Western blot.

### AMPK activity assay

HUVEC stably expressing EV or FLAG-K1 were incubated in EBM-2 without serum and growth factors for 24 hours. Either 5 uM compound C or DMSO (0.05%) control was added at the start of starvation. Cells were washed with cold PBS and then lysed with a buffer containing 50mM Tris-HCL pH 7.4, 1 mM EDTA, 1 mM EGTA, 250 mM mannitol, 1% Triton X-100 and proteinase (Roche) and phosphatase inhibitors (Roche). Lysates were then clarified by centrifugation. For the AMPK activity assay, reagents were purchased from SignalChem and the manufacturer’s protocol followed. Briefly, 10 μLs of cell lysate was incubated with 5 μL of 1 mg/mL SAMS or peptide substrate solution, 5 μLs 0.5 mM AMP solution and 5 μLs γ-32P-ATP assay cocktail. Gamma-32P-ATP was purchased from Perkin Elmer. The mixture was incubated at room temperature for 30 minutes and then 20 μLs was added to phosphocellulose paper and washed 2 times in 1% phosphoric acid solution. Counts per minute (cpm) were acquired using a PerkinElmer liquid scintillation analyzer.

### Immunofluorescence and confocal microscopy

Approximately 120,000 HUVEC cells stably expressing EV or FLAG-K1 were plated in MatTek 35 mm glass-bottom dishes. Cells were washed with PBS and fixed by 15-minute incubation in 3.7% formaldehyde at room temperature. Cells were washed 3X with PBS and then permeabilized by 15-minute incubation at room temperature in 0.2% Triton-X 100/PBS. Cells were then washed again 3X with PBS and then blocked in 10% bovine serum albumin (BSA) PBS for 30 minutes. Cells were stained 1:200 with a directly conjugated FITC-ECS (DDDDK) polyclonal antibody (Bethyl laboratories) and anti-AMPKβ1/2 (1:50, Cell Signaling) in 10% BSA for 1 hour at room temperature. Cells were washed 2X quick followed by 3X 5-minute washes. Samples were then incubated with anti-rabbit Alexa Fluor 647 (1:600) secondary antibody in 10% BSA at room temperature for 1 hour. All samples were then stained with DAPI for 1 minute and washed. Fluorescent images were acquired by taking z-stacks using a 63X oil objective on a Zeiss 700 confocal microscope. The overlap coefficient according to Manders (R) was determined using Image J. Confocal images from 5 cells in their entirety were evaluated for co-localization by calculating the overlap coefficient.

### Generation of WT or mutant K1 recombinant viruses

The construction of the KSHV WT and mutant recombinant viruses has previously been described in detail [46]. Briefly, the BAC 16 was kindly provided by Dr. Jae U. Jung. pcDNA3 WT and mutant K1 constructs were used as templates for construction of recombinant viruses. pcDNA3 WT K1_FLAG_, which has a FLAG tag at the N-terminus, was constructed as previously described [68]. The pcDNA3-K1_5XSTOP_ construct has 3 stop codons following the start codon of WT K1 FLAG and two TGA stop codons replacing ATG start codons at positions 481 and 763. K1_5XSTOP_ was inserted into BamHI and EcoRI sites of pcDNA3 WT K1_FLAG_. The original K1 gene is located within the BAC16 genome at position 105 to 959. KSHV-K1_REV_ was made by replacing the mutant K1 gene from KSHV-K1_5XSTOP_ with a RpsL-Neo cassette and then replaced the RpsL-Neo cassette with WT K1_FLAG._ The recombinant viruses containing KSHV-K1_WT_, KSHV-K1_5XSTOP_, KSHVΔK1 and KSHV-K1_REV_ were made utilizing the Red/ET recombination system (Gene Bridges Inc) as per the manufacturer’s instructions. KSHVΔK1 was constructed by replacing the K1 gene with the linear RpsL-neo cassette that is flanked by homologous arms [46].

### Establishment of cells stably infected with WT or mutant K1 recombinant viruses

Five × 10^5^ cells of WT or recombinant virus infected iSLK cells were plated in one well of a 6- well plate overnight after which cells were reactivated with 3 μg/ml doxycycline and 1 mM sodium butyrate for 3 days. Supernatant was collected and cleared by centrifugation at 950g for 10 min and filtered through a 0.45 μm filter. iSLK cells were infected as previously described [46] and maintained in the presence of 1 μg/ml puromycin, 250 μg/mL G418, and 1.2 mg/mL hygromycin [46]. In order to infect HUVEC, the filtered viral supernatants from reactivated iSLK cells were incubated with the immortalized HUVEC cells in the presence of 8 μg/ml of polybrene and centrifuged for 2 hours at 3000 RCF at 30°C. The cells were then placed in an incubator with 5% CO_2_ at 37°C. At 48 hours post-infection, 30 μg/ml hygromycin was added to the media to select for HUVEC stably infected with KSHV-K1_WT_, KSHV-K1_5XSTOP_, KSHVΔK1 or KSHV-K1_REV_ (revertant) recombinant viruses. The infected HUVEC cells were also maintained in the presence of 30 μg/ml hygromycin.

## Supporting Information

S1 Fig Cell viability following starvation and then nutrient replenishment. (A) Cell viability was determined by trypan blue exclusion assay in HUVEC infected with KSHV-K1_WT_, KSHV-K1_5XSTOP_, KSHVΔK1, and KSHV-K1_REV_ following serum and growth factor starvation for 72 hours. (B) HUVEC infected with KSHV-K1_WT_, KSHV-K1_5XSTOP_, KSHVΔK1, and KSHV-K1_REV_ were deprived of serum and growth factors for 72 hours. At 72 hours nutrients were replenished. Cell viability was determined by trypan blue exclusion assay after 72 hours post nutrient replenishment.
(TIF)

S2 Fig Reduced cell viability in HUVEC stably infected with KSHV-K1_WT_ following treatment with AMPKα1/AMPKα2 siRNA compared to KSHV-K1_WT_ treated with non-specific siRNA (NS). HUVEC cells were transiently transfected with AMPKα1 and AMPKα2 siRNA or non-specific (NS) siRNA. (A) Cell viability was evaluated by determining ATP levels using an ATP luminescence assay (Promega) at 48 and 72 hours post transfection. (B) AMPKα1 and AMPKα2 expression in the lysates from (A) at 72 hours post transfection was determined by SDS-PAGE and Western blot. Student’s t test, *P<0.05.
(TIF)

S3 Fig Mass Spectrometry of K1 interacting proteins. **A)** Stable cell lines expressing a FLAG and HA double epitope-tagged version of K1 and EV HEK-293 cells were subjected to tandem affinity purification. FLAG-HA-K1 or EV HEK-293 expressing cells were lysed and the lysates were incubated with anti-FLAG M2 affinity gel, washed and eluted with 3X FLAG peptide. The eluates were subsequently incubated with an anti-HA resin, washed with NP40 buffer and eluted. The eluates were resolved by sulfate polyacrylamide gel electrophoresis (SDS-PAGE) and Coomassie stained. B) The eluates from (A) were also subjected to SDS-PAGE and Western blot analysis with an anti-FLAG antibody to detect the presence of the monomer and oligomeric forms of K1.
(TIF)
