# Supplementary figures and images for: The KSHV K1 Protein Modulates AMPK Function to Enhance Cell Survival
Source: PLoS Pathog. 2016 Nov 9;12(11):e1005985. doi: 10.1371/journal.ppat.1005985 (PMC5102384; doi:10.1371/journal.ppat.1005985)

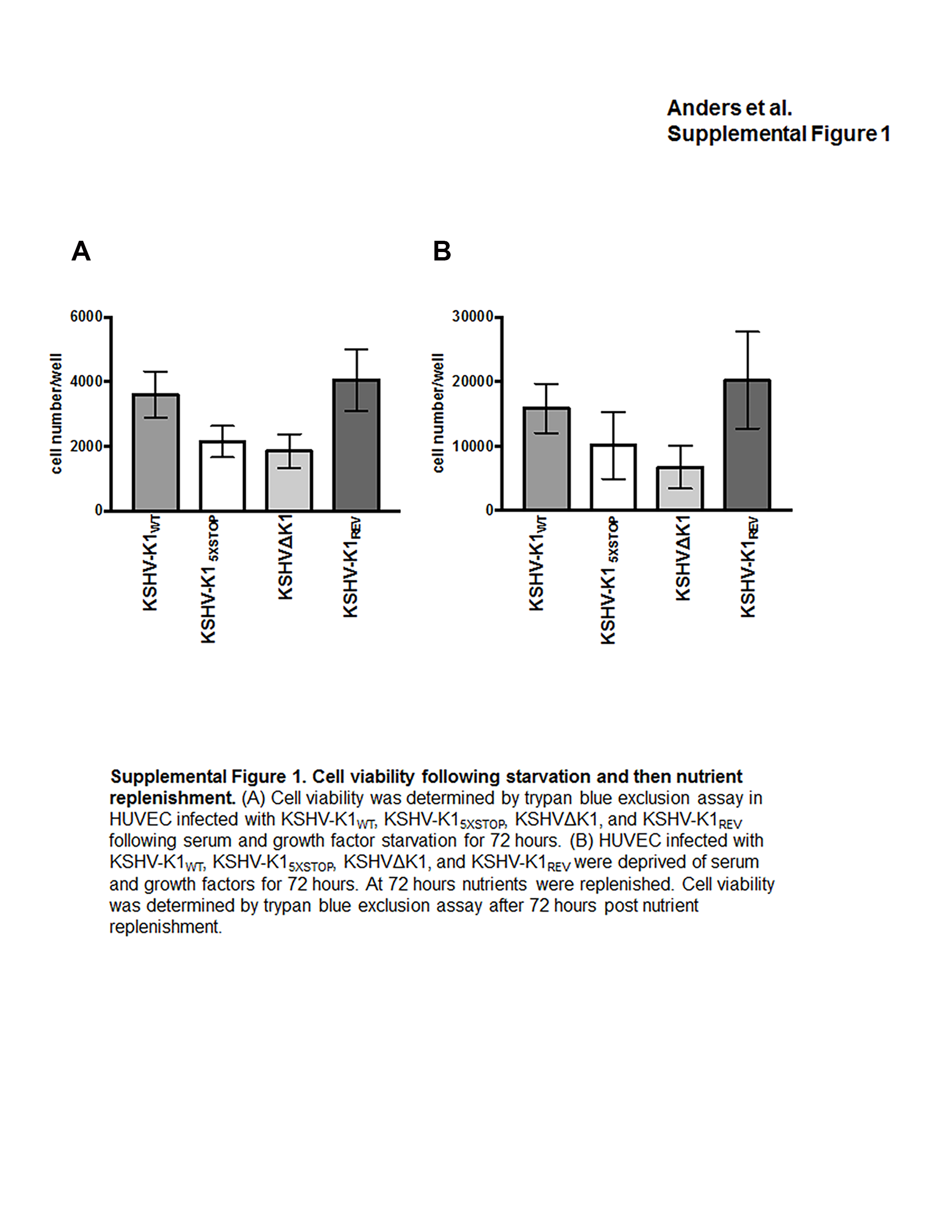

Supplement: S1 Fig — (A) Cell viability was determined by trypan blue exclusion assay in HUVEC infected with KSHV-K1WT, KSHV-K15XSTOP, KSHVΔK1, and KSHV-K1REV following serum and growth factor starvation for 72 hours. (B) HUVEC infected with KSHV-K1WT, KSHV-K15XSTOP, KSHVΔK1, and KSHV-K1REV were deprived of serum and growth factors for 72 hours. At 72 hours nutrients were replenished. Cell viability was determined by trypan blue exclusion assay after 72 hours post nutrient replenishment. (TIF) [file ppat.1005985.s001.tif]

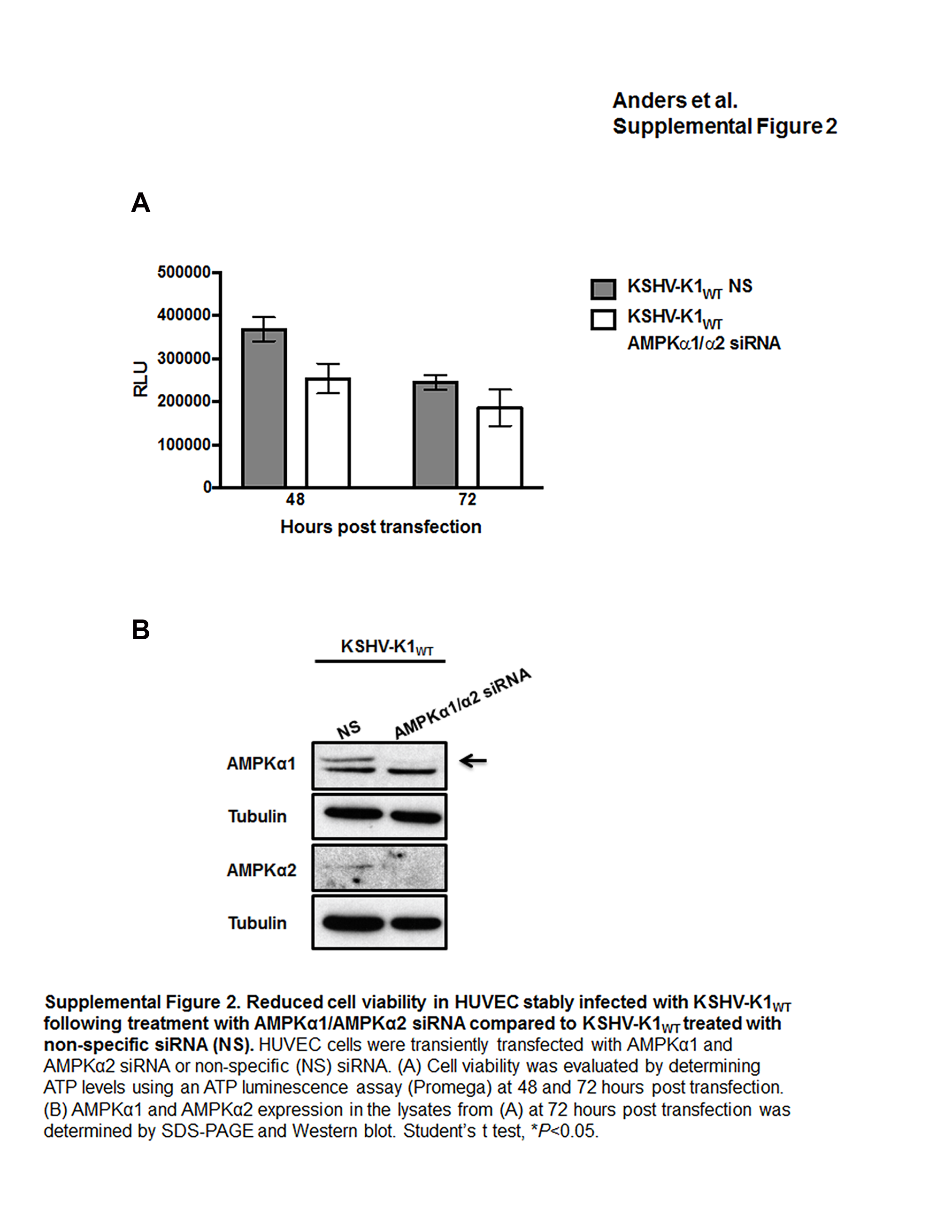

Supplement: S2 Fig — HUVEC cells were transiently transfected with AMPKα1 and AMPKα2 siRNA or non-specific (NS) siRNA. (A) Cell viability was evaluated by determining ATP levels using an ATP luminescence assay (Promega) at 48 and 72 hours post transfection. (B) AMPKα1 and AMPKα2 expression in the lysates from (A) at 72 hours post transfection was determined by SDS-PAGE and Western blot. Student’s t test, *P<0.05. (TIF) [file ppat.1005985.s002.tif]

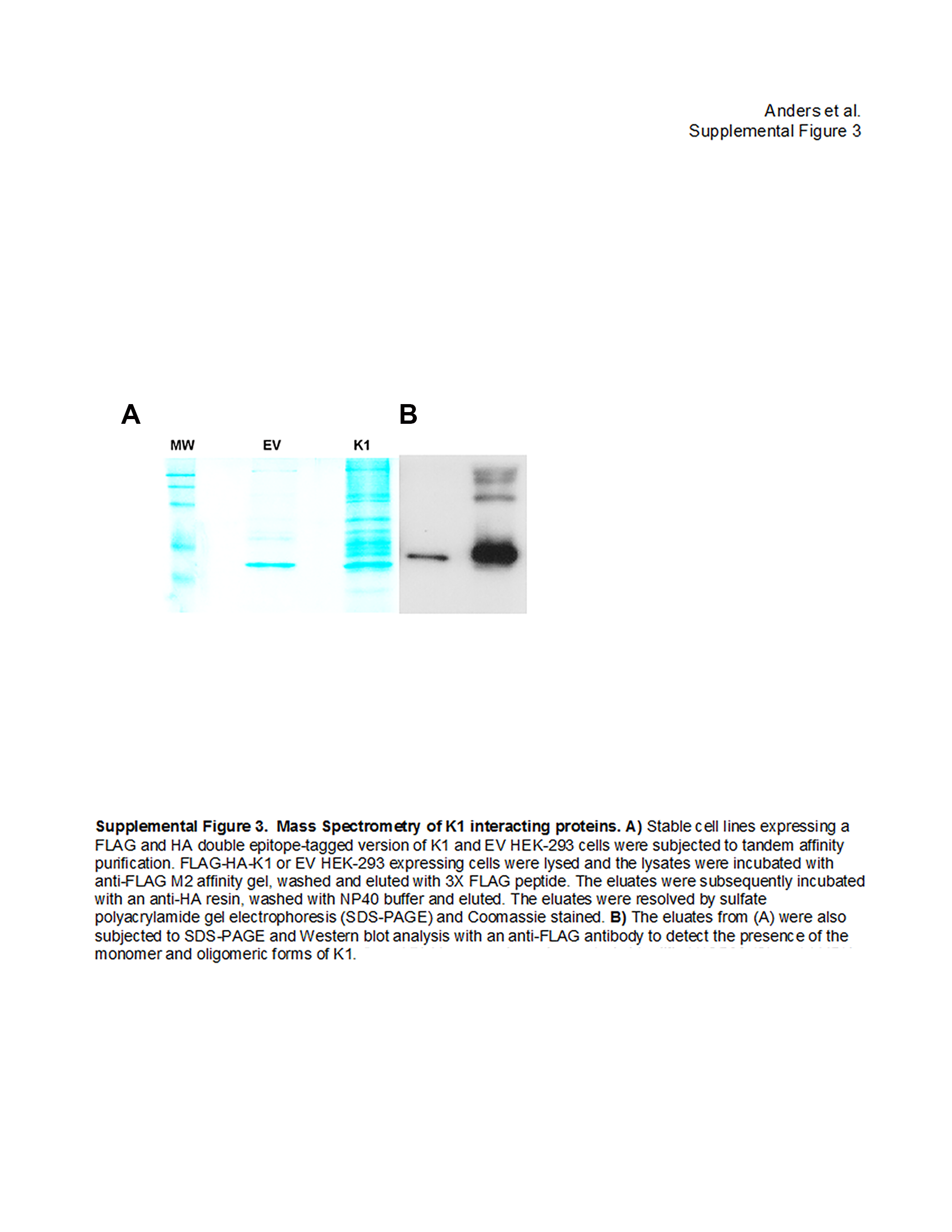

Supplement: S3 Fig — A) Stable cell lines expressing a FLAG and HA double epitope-tagged version of K1 and EV HEK-293 cells were subjected to tandem affinity purification. FLAG-HA-K1 or EV HEK-293 expressing cells were lysed and the lysates were incubated with anti-FLAG M2 affinity gel, washed and eluted with 3X FLAG peptide. The eluates were subsequently incubated with an anti-HA resin, washed with NP40 buffer and eluted. The eluates were resolved by sulfate polyacrylamide gel electrophoresis (SDS-PAGE) and Coomassie stained. B) The eluates from (A) were also subjected to SDS-PAGE and Western blot analysis with an anti-FLAG antibody to detect the presence of the monomer and oligomeric forms of K1. (TIF) [file ppat.1005985.s003.tif]
